# Within‐person biological mechanisms of mood variability in childhood and adolescence

**DOI:** 10.1002/hbm.26766

**Published:** 2024-07-24

**Authors:** Yara J. Toenders, Marleen H. M. de Moor, Renske van der Cruijsen, Kayla Green, Michelle Achterberg, Eveline A. Crone

**Affiliations:** ^1^ Developmental and Educational Psychology Leiden University Leiden The Netherlands; ^2^ Leiden Institute for Brain and Cognition Leiden University Leiden The Netherlands; ^3^ Erasmus School of Social and Behavioral Sciences Erasmus University Rotterdam Rotterdam The Netherlands; ^4^ Department of Psychology, Education and Child Studies Erasmus University Rotterdam Rotterdam The Netherlands; ^5^ Behavioral Science Institute Radboud University Nijmegen Nijmegen The Netherlands

**Keywords:** adolescence, mood variability, puberty, random intercept cross‐lagged panel models, resting‐state fMRI, testosterone

## Abstract

Mood variability, the day‐to‐day fluctuation in mood, differs between individuals and develops during adolescence. Because adolescents show higher mood variability and average mood than children and adults, puberty might be a potential biological mechanism underlying this increase. The goal of this preregistered developmental study was to examine the neural and hormonal underpinnings of adolescent‐specific within‐person changes in mood variability, with a specific focus on testosterone, cortisol, pubertal status, and resting‐state functional brain connectivity. Data from two longitudinal cohorts were used: the L‐CID twin study (aged 7–13, *N* at the first timepoint = 258) and the accelerated Leiden Self‐Concept study (SC; aged 11–21, *N* at the first timepoint = 138). In both studies resting‐state functional magnetic resonance imaging (rs‐fMRI) data was collected, as well as daily mood. Additionally, in the SC study self‐reported puberty testosterone and cortisol were collected. Random intercept cross‐lagged panel models (RI‐CLPM) were used to study the within‐person relations between these biological measures and mood variability and average mood. Mood variability and average mood peaked in adolescence and testosterone levels and self‐reported puberty also showed an increase. Connectivity between prefrontal cortex (dlPFC and vmPFC) and subcortical regions (caudate, amygdala) decreased across development. Moreover, higher testosterone predicted average negative mood at the next time point, but not vice versa. Further, stronger vmPFC–amygdala functional connectivity predicted decreases in mood variability. Here, we show that brain connectivity during development is an important within‐person biological mechanism of the development of mood in adolescents.

**Practitioner Points:**

- Mood variability peaks in adolescence.
- Within‐person changes in testosterone predict within‐person changes in mood.
- Within‐person changes in vmPFC–amygdala connectivity predict within‐person changes in mood variability.

## Graphical abstract

Both mood variability and cortico‐subcortical brain connectivity develop during adolescence, with mood variability showing a peak and vmPFC–amygdala connectivity decreasing. We showed that brain connectivity during development is an important within‐person biological mechanism of the development of mood in adolescents.

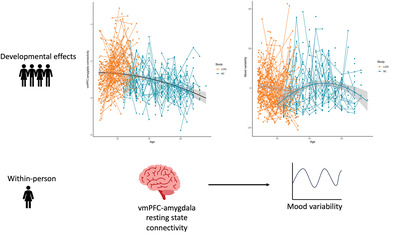

## 1 INTRODUCTION

Mood variability, which is defined as the day‐to‐day fluctuation in positive and negative mood states, is an important psychological state that differs between individuals and changes with developmental stages. Understanding mood variability is important as specifically negative mood variability is often associated with increased feelings of anxiety and depression and precedes future symptoms of anxiety and depression (Maciejewski et al., 2014; Toenders et al., 2022). Prior research showed that negative mood variability shows a peak in adolescence, possibly reflecting a period in which more day‐to‐day fluctuation in negative mood is more prevalent (Maciejewski et al., 2015; Toenders et al., 2022). This peak is specifically strong in females in mid‐adolescence (Toenders et al., 2022). Average negative mood shows a peak in the same period and mood disorders often have their onset in adolescence (Kessler et al., 2005) and are more prevalent among females (Merikangas et al., 2009). The goal of this developmental study was to examine the neural and hormonal underpinnings of adolescent‐specific within‐person changes in mood variability, with a specific focus on testosterone, cortisol, pubertal status, and resting‐state functional brain connectivity.

Since adolescents show higher mood variability than children and adults (Miller Buchanan et al., 1992), a plausible biological mechanism is that mood variability is associated with puberty. The increase in testosterone in males and females starts around age 10 and peaks around age 20, with an especially large increase in males (Handelsman et al., 2016; Kelsey et al., 2014). Testosterone has repeatedly been found to be associated with mood using between‐person designs, for example testosterone concentration in adults correlates negatively with reaction time when recognizing fearful faces (Derntl et al., 2009), and prior research reported an association between testosterone levels, anger, and tension in young adults (Copeland et al., 2019; van Honk et al., 1999). Some inconsistencies about the directionality between testosterone and mood between sexes have been reported, with increases in testosterone in both sexes being associated with elevated anxiety (Chronister et al., 2021); increases in testosterone in girls specifically were associated with depression (Copeland et al., 2019), but in contrast, in boys lower testosterone was also associated with higher anxiety and depression (Granger et al., 2003). Simultaneously, cortisol release also increases during puberty (Miller et al., 2016; Romeo, 2005). The hypothalamus–pituitary–adrenal (HPA) axis that leads to the secretion of cortisol matures during adolescence (Marceau et al., 2015). Prior research has shown between‐person positive associations between cortisol and mood variability in adults (Human et al., 2015; Simpson et al., 2008). Taken together, testosterone (Handelsman et al., 2016), cortisol levels (Marceau et al., 2015), and mood variability and average mood (Toenders et al., 2022) increase in adolescence and show between‐person associations, leading to the hypothesis that hormone changes in adolescence might be biological precursors of mood variability. However, this hypothesis has not yet been examined empirically using a within‐person longitudinal design ranging from late childhood to early adulthood.

In addition to hormonal changes, mood variability has previously been associated with neural changes, such as alterations in dorsolateral prefrontal cortex (dlPFC) thickness and connectivity (Toenders et al., 2022; Votinov et al., 2020). Successful emotion regulation, which may be involved in mood variability, has been found to be associated with lesser amygdala—dlPFC connectivity using resting state fMRI (rs‐fMRI) (Uchida et al., 2014). However, the dynamics between neural changes and mood variability are not yet well understood. It has been hypothesized that sex steroids, such as testosterone, do not only affect brain activation, but also influence brain organization during puberty (Liao et al., 2021; Peper et al., 2011; Wierenga et al., 2018). For example, prior research showed that higher levels of testosterone in puberty were associated with reduced amygdala—orbitofrontal cortex (OFC) resting state connectivity (Peters et al., 2015; Spielberg et al., 2013). Exogenous testosterone has been shown to reduce amygdala—dlPFC connectivity in both directions in men and amygdala—OFC connectivity in women (van Wingen et al., 2010; Votinov et al., 2020). Cortisol has also been shown to be associated with brain connectivity, such that endogeneous cortisol was associated with increased negative amygdala–medial prefrontal cortex (mPFC) connectivity (Veer et al., 2012). Developmental research has further shown that connections between subcortical and cortical regions strengthen and de‐strengthen with age (van Duijvenvoorde et al., 2016). Notably, the decrease in connectivity between ventromedial prefrontal cortex (vmPFC) and subcortical regions was better explained by pubertal development than age (van Duijvenvoorde et al., 2019), suggesting that gonadal hormones may affect neural connectivity. Examining the functional connectivity between the amygdala and the prefrontal cortex might therefore provide more insight into the neural mechanisms of mood variability and average mood. This functional connectivity could be a potential brain correlate of pubertal‐driven changes in mood variability and average mood.

The current preregistered study aimed to elucidate the development of mood and mood variability during adolescence, by examining the within‐person associations between subcortical–cortical connectivity, hormone levels, and mood variability and average mood in two large longitudinal cohorts: the Leiden Consortium on Individual Development (L‐CID) and the Leiden Self‐Concept Study (SC) (Crone et al., 2020, 2022). We included L‐CID and SC to assess the robustness of the findings across two cohorts; the first examining the transition from middle childhood to early adolescence (7–14 years) and the second examining the transition from late childhood/early adolescence to young adulthood (11–24 years). First, we hypothesized that an increase in testosterone, cortisol, and pubertal status would precede mood variability increase from childhood to adolescence. Additionally, increased resting state connectivity between the dlPFC and amygdala was hypothesized to represent advanced development, and therefore is expected to precede lower mood variability from mid‐adolescence to young adulthood (van Duijvenvoorde et al., 2016).

To assess the within‐person associations between mood variability/average mood and brain connectivity and hormone levels, random intercept cross‐lagged panel models (RI‐CLPM) were used. Since youth show individual differences in mood and biological risk factors (Handelsman et al., 2016; Maciejewski et al., 2015), the RI‐CLPM captures stable between person differences and models the within‐person associations between the biological risk factors and mood variability. This allowed us to study the within‐person longitudinal relation between mood and biological processes.

## 2 METHODS

### 2.1 Participants

**FIGURE 1:**
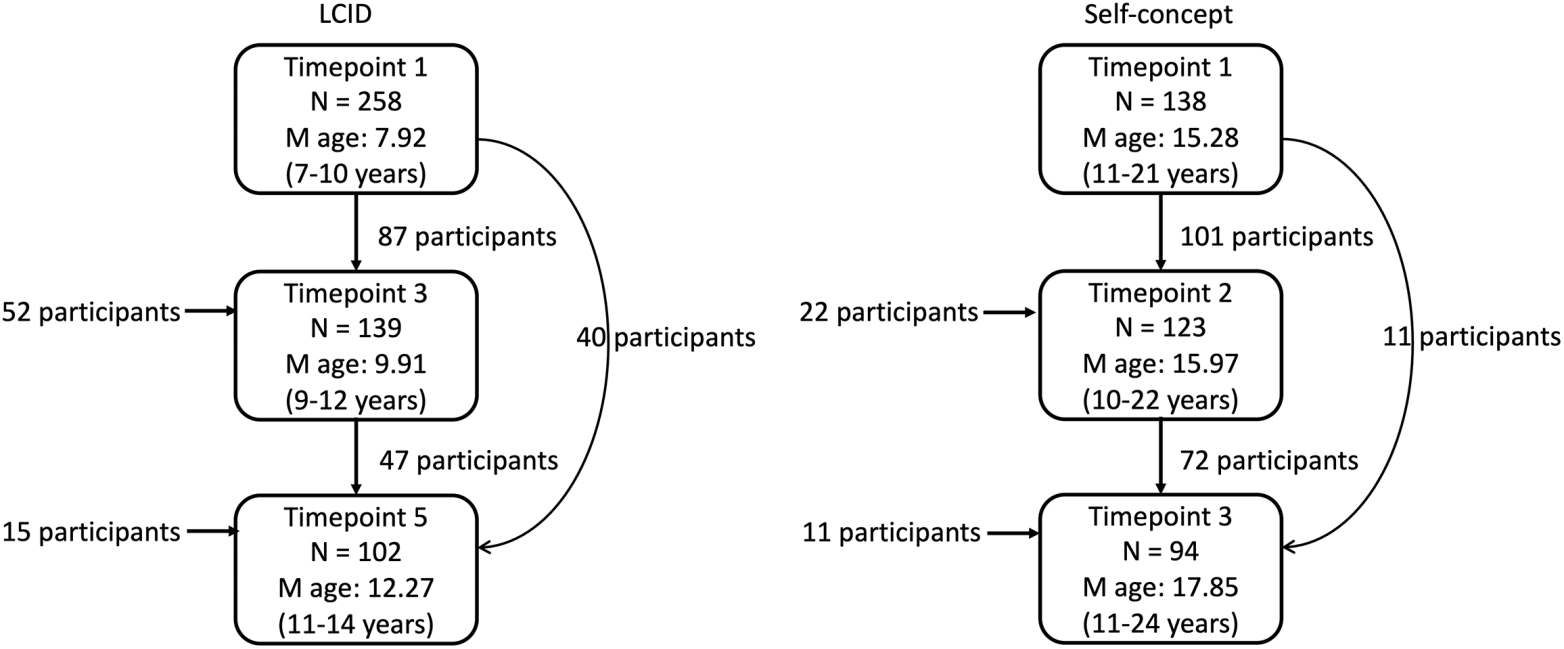
Flowchart of participants. 258 participants were included at timepoint 1 of the L‐CID study, 139 at timepoint 3 (of which 87 participants who also participated at T1 and 52 new participants), and 102 at T5 (of which 47 also participated at T3, 40 participated at T1 but not T3 and 15 new participants). In the SC study 138 participants were included at T1, 123 at T2 (of which 101 participants who also participated at T1 and 22 new participants), and 94 at T3 (of which 72 also participated at T2, 11 participated at T1 but not T2 and 11 new participants).

Participants from the L‐CID and SC cohorts were included (Figures 1, S1, and S2, Supporting Information). The L‐CID study consists of an early and middle childhood cohort; for this study the middle childhood cohort from L‐CID was included. L‐CID is a cohort‐sequential longitudinal study, including twins aged 7–9 years at the first wave in 2015, who were followed every 2 years for three waves in total (Crone et al., 2020; Euser et al., 2016).

Same‐sex twins were recruited through municipalities registries. Inclusion criteria were born between 2006 and 2009, living in the Western municipalities of the Netherlands, being fluent in Dutch and normal (or corrected to normal) vision. Every 2 years, participants came to the lab accompanied by their primary parent to undergo an MRI scan. In between these MRI timepoints, a home visit took place. However, for this study only the three MRI timepoints were used: T1 (*N* = 258), T3 (*N* = 139), and T5 (*N* = 102). Following each MRI timepoint, the primary caregiver filled out daily questionnaires for four consecutive days, except at timepoint 5, this was only done for 2 days. The study was approved by the Dutch Central Committee on Research Involving Human Subjects (CCMO, number NL50277.058.14). Written informed consent was signed by both parents.

The SC study has an accelerated longitudinal design including adolescents aged 11–24 years, who were followed every year for three waves in total, starting in 2016. Healthy, right‐handed adolescents participated in the study and were recruited from schools and online. Participants were excluded if they were diagnosed with a neurological or psychiatric disorder at the first timepoint. At baseline participants were aged 11–21 years, and they were followed annually for three timepoints. At each visit MRI measures, hormone measures and questionnaire data were collected. The SC study was approved by the Medical Ethics Committee (CME) of the Leiden University Medical Centre (LUMC). Written informed consent was signed by participants and the parents of minors.

### 2.2 Behavioral measures

Daily mood was examined in age‐specific ways, and thus relied on parent‐report for the L‐CID study and on self‐report for the SC study.

#### 2.2.1 Mood assessment in the L‐CID study

Primary caregivers were prompted four times a day to fill out three questions on their child's mood (at 9 am, 11 am, 3 pm, and 7 pm). They could rate these questions on a scale from 0 to 100. The questions were one question on the energy level of the child (0 was tired, 100 awake), one on mood of the child (0 unhappy, 100 happy), and one on how tense the child was (0 tense, 100 relaxed). The variables were recoded, so for each item 100 was positive mood and 0 negative mood. This was filled out daily for four consecutive days (two weekdays and two weekend days) for the first two timepoints, and two weekend days for the last timepoint due to time constraints. The terminology used throughout the 4 days was alternated. For consistency among the cohorts, only the data from the last time point of the day (7 pm) was used. Mood variability was calculated per question as the absolute difference between successive days. These daily difference scores were averaged over the number of consecutive days the caregiver rated the child's mood. The variability per question was summed across the three questions to create a total mood variability score. These scores were normalized to create a score between 0 and 1. Higher total mood variability indicated larger fluctuations in mood across days (mean correlation averaged over subscales and waves: 0.21).

#### 2.2.2 Mood assessment in the SC study

In the SC study, adolescents filled out the profiles of mood states (POMS) for five consecutive days after the lab visit (Curran et al., 1995). The POMS consists of 32 adjectives that were rated on a 5‐point Likert scale to which degree the adjective describes the adolescent's current mood. It consists of five subscales: anger, depression, fatigue, tension, and vigor. The tension, fatigue, and depression items were similar to the items used in LCID. In the SC study, participants received a notification at 6 pm and responded to these adjectives in an online questionnaire. Mood variability was calculated using only the first four negative subscales, in line with prior research, since vigor shows a different development compared to the negative mood subscales (Toenders et al., 2022). Only adolescents with mood ratings on three or more consecutive days were included. Items were averaged to calculate the subscale score. Similar to in L‐CID, the absolute difference between consecutive days was calculated per subscale. Next, this was averaged over the days that mood was reported and the four subscales were summed to create a total mood variability score. This score was normalized, to be able to compare the score to the parent‐reported scores in L‐CID. Additionally, average mood was calculated by summing the averaged negative subscales. Outliers in average mood were winsorized at 0.05 and 0.95 cut‐off points. If participants had fewer than 10 single items missing on the mood questionnaire in the SC study, the data was imputed using predictive mean matching in the “mice” package in R (Van Buuren & Groothuis‐Oudshoorn, 2011). The subscales in the current sample show good reliability (Cronbach's *α* = 0.84, *α* = 0.89, *α* = 0.87, *α* = 0.89, and *α* = 0.82, for tension, depression, anger, fatigue, and vigor, respectively). Both the SC and LCID study were included in mood variability study. The analyses for average mood are based on data from the SC study. For these analyses, data from the LCID study were not included because in the LCID study positive and negative emotions were intermixed, which does not allow for a sum score of average mood.

### 2.3 Hormone measures

Hormone measures were only available for the SC study. Below we report how assessment took place for testosterone levels, cortisol levels, and pubertal status. No other pubertal hormone measures were available in the SC study.

#### 2.3.1 Testosterone

Testosterone levels were measured in morning saliva. Samples were collected in the morning by passive drool before breakfast/tooth brushing. The samples were assayed at ARU Biomarker Laboratory, Cambridge, UK using an enzyme‐linked immunoassay (ELISA). Testosterone was measured in duplicate, and the lower detection limit was 4 pg/mL. The intra‐ and interassay variability were 3.7% and 5.9%, respectively. Testosterone was normalized per wave to examine testosterone compared to the testosterone levels of other participants to correct for batch effects.

#### 2.3.2 Cortisol

Cortisol levels were also measured in morning saliva. Samples were collected by passive drool. The samples were also assayed by ARU Biomarker Laboratory, Cambridge, UK using ELISA kits. Cortisol was measured in duplicate, and the lower detection limit was 0.03 μg/dL. The intra‐ and inter‐assay variability were 4.3% and 4.2%, respectively. Cortisol was normalized per wave to examine testosterone compared to other participants to correct for batch effects.

#### 2.3.3 Pubertal status

Pubertal status was measured using the self‐report Pubertal Development Scale (PDS) (Petersen et al., 1988). Participants rate their secondary sexual characteristics (e.g., body hair, breast development for females) on a 4‐point Likert scale. The average score (between 1 and 4) was used in the analyses. Timepoints of participants that showed a decrease in pubertal status were excluded (8.8%).

### 2.4 MRI acquisition

MRI scans for both studies were obtained at the same scan site, on a Philips Ingenia 3.0 Tesla MR scanner. A standard whole‐head coil was used. First, a functional resting state scan was obtained (TR = 2.2 s, TE = 30 ms, flip angle = 80°, 37 slices, voxel size = 2.75 × 2.75 × 2.75 mm, FOV = 220 × 220 × 111.65 mm). During the acquisition of the resting state scan, participants were instructed to look at a fixation cross. Next, a high‐resolution T1 was collected (TR = 9.72 ms, TE = 4.95 ms, flip angle = 8°, 140 slices, voxel size = 0.875 × 0.875 × 0.875 mm, FOV = 224 × 178.5 × 168 mm).

### 2.5 fMRI preprocessing

Resting state MR images were processed in HALFpipe version 1.2.2 (Waller et al., 2021). HALFpipe is a containerized pipeline to process fMRI scans and uses fMRIprep (Esteban et al., 2019). Standardized protocols were used to increase reproducibility and harmonization analyses and quality control (https://enigma.ini.usc.edu/protocols/functional-protocols/). Pre‐processing included denoising (ICA‐AROMA with 6 motion parameters), motion correction, distortion correction, spatial smoothing, grand mean scaling, temporal filtering and spatial normalization, and registration to the MNI152 template. Manual quality control was done to assess data quality, temporal signal to noise ratio, excessive movement, and quality of registration to the T1 image. Participants were excluded if they did not pass manual quality control, if there was excessive head motion (any rotation >4 mm or average FD >0.3 mm), and if their MR image covered less than 80% of the seed (497 images were excluded in total, mostly due to movement by young children).

### 2.6 fMRI processing

Seed based connectivity analyses were performed using FSLs GLM function within HALFpipe. The seeds of interest were selected of emotion regulation maps on Neurosynth (http://neurosynth.org/; derived July 2022). The center of two regions involved in emotion regulation (bilateral dlPFC and vmPFC) were derived from these maps. A 6 mm sphere was created around these centers. In addition, anatomical masks of the subcortical regions were used (amygdala and caudate) to examine the connectivity between cortical and subcortical regions.

### 2.7 Statistical analyses

All analyses were conducted with R, using the *lme4* and *lavaan* packages (Bates et al., 2015; Rosseel, 2012). First, descriptives were calculated for all outcome measures per study, as well as the correlations across timepoints. Next, the correlations between biological and mood variables were investigated per study across timepoints. Next, the development of mood variability across both cohorts and average mood in the SC study was examined using linear and quadratic association with age by sex in a linear mixed model. The development of the pubertal measures was studied in the SC cohorts using linear mixed models to examine the linear and quadratic association with age by sex. The same was done for the brain connectivity measures in the combined sample.

#### 2.7.1 Random‐intercept cross‐lagged panel models

The data from the SC study was used to examine the within‐person associations between mood variability/average mood and hormone levels/pubertal status, and the combined sample of the SC and L‐CID data was used to study the within‐person associations between resting‐state connectivity and mood variability.

RI‐CLPMs were used to test the bidirectional within‐person relations between hormone levels/pubertal status, resting‐state connectivity, and mood variability/average mood (Hamaker et al., 2015). Random intercepts capture the stable between‐person differences. Further, within‐person autoregressive effects represent the within‐person stability (over timepoints) of mood variability, hormone levels and resting‐state connectivity. To examine the within‐person relations between hormones or brain connectivity and mood variability/average mood, the cross‐lagged effects were tested. Sex was added as a time‐invariant covariate. In RI‐CLPMs including the L‐CID study, family was also included as time‐invariant covariate, since twin pairs were nested within families. In total, seven bivariate models (testosterone, cortisol, PDS, dlPFC–amygdala, dlPFC–caudate, vmPFC–amygdala, and vmPFC–caudate connectivity) were run with mood variability and three models with average mood (testosterone, cortisol, PDS).

In a next step, trivariate RI‐CLPMs were estimated to test whether the relations between puberty, testosterone/cortisol at timepoint 1 and mood variability at timepoint 3 were mediated by resting state connectivity at timepoint 2, or vice versa (Fredrick et al., 2021). In total 12 models were run with testosterone, cortisol or PDS as predictor, dlPFC–amyg, dlPFC–caudate, vmPFC–amygdala, or vmPFC–caudate connectivity as mediator and mood variability as outcome.

The following criteria were used to assess model fit: *χ* ^2^ (*p* < 0.05), Comparative Fit Index (CFI) > 0.90 and root‐mean‐square error of approximation (RMSEA) < 0.08 (adequate fit). Paths were constrained to be equal across time points for the SC study because of its accelerated design, meaning that participants had different ages at the timepoints. Next, model fit of the RI‐CLPM was compared to the nested classical cross‐lagged panel model (CLPM) that does not consider within person relations, but only between person relations.

## 3 RESULTS

The demographic variables per cohort per wave are displayed in Table 1. The correlation of outcome measures between timepoints and stability of these measures are displayed in Tables S1 and 2. The correlation matrices between outcome variables are displayed in Figures S3 and S4.

**TABLE 1:** Demographic characteristics per study per timepoint.

	L‐CID			SC		
Timepoint	1	3	5	1	2	3
*N*	258	139	102	138	123	94
Age	7.92 (0.66)	9.91 (0.69)	12.27 (0.66)	15.28 (2.84)	15.97 (3.22)	17.85 (3.43)
Sex, *N* female (%)	130 (50%)	71 (51%)	50 (49%)	76 (55%)	62 (50%)	58 (62%)
Puberty						
Testosterone				110.64 (74.25)	64.78 (56.41)	159.30 (87.66)
Cortisol				0.34 (0.18)	0.26 (0.13)	0.33 (0.20)
PDS				2.89 (0.82)	3.09 (0.81)	3.28 (0.72)
Brain connectivity^a^						
dlPFC to amygdala connectivity	0.96 (0.82)	0.87 (1.04)	1.23 (1.16)	0.61 (0.74)	0.59 (0.71)	0.55 (0.60)
dlPFC to caudate connectivity	1.82 (1.54)	2.36 (1.54)	2.90 (1.74)	2.19 (1.51)	1.78 (1.19)	1.86 (1.07)
vmPFC to amygdala connectivity	1.15 (0.85)	2.47 (1.67)	3.02 (1.71)	0.72 (0.77)	0.76 (0.73)	0.72 (0.75)
vmPFC to caudate connectivity	1.94 (1.35)	1.65 (1.15)	1.54 (0.91)	1.75 (1.38)	1.37 (1.12)	1.33 (1.12)
Mood						
Mood variability	64.01 (25.42)	59.71 (28.43)	59.60 (37.69)	8.41 (5.72)	8.93 (6.29)	9.39 (6.02)
Average mood	195.83 (29.70)	188.39 (31.23)	191.36 (35.06)	10.82 (10.67)	12.91 (11.34)	13.54 (10.73)

*Note*: Mean (SD) are being displayed. Data from 325 unique participants from the LCID study were included, leading to 499 observations. Data from 171 participants from the SC study were included, leading to a total of 355 observations.

^a^ Parameter estimates.

**TABLE 2:** Stability of the outcome measures in combined sample.

Measure	ICC [95%CI]
Mood variability	0.16 [0.05–0.26]
Connectivity dlPFC–amygdala	0.24 [0.11–0.26]
Connectivity dlPFC–caudate	0.31 [0.15–0.30]
Connectivity vmPFC–amygdala	0.34 [0.24–0.42]
Connectivity vmPFC–caudate	0.36 [0.27–0.45]

### 3.1 Development of mood variability

**FIGURE 2:**
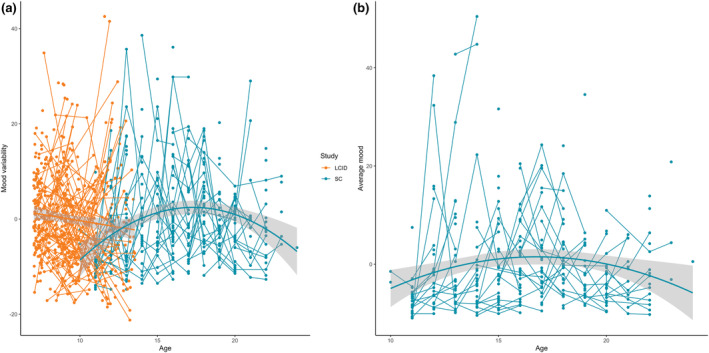
Development of mood variability (left) and average mood (right) across age in the L‐CID and SC study. Mood variability showed no age effect in L‐CID and a quadratic age effect in SC, and average mood showed a quadratic age effect in the SC study.

The developmental trajectories of mood variability are reported separately for the L‐CID and SC cohort (Figure 2). No age effect for mood variability was found in the L‐CID (*F*(381.68) = 3.40, *p* = 0.07) and a quadratic age effect for mood variability was found in the SC study with a peak in mid adolescence (*F*(310.46) = 19.13, *p* < 0.001). When examining the interaction between age and sex on mood variability, no interaction effect was found in the L‐CID study (*F*(380.53) = 1.63, *p* = 0.20). Average negative mood showed a quadratic age effect in the SC study, with a peak in mid adolescence (*F*(276.15) = 8.97, *p* = 0.003).

### 3.2 Development of testosterone, cortisol, and self‐reported puberty

**FIGURE 3:**
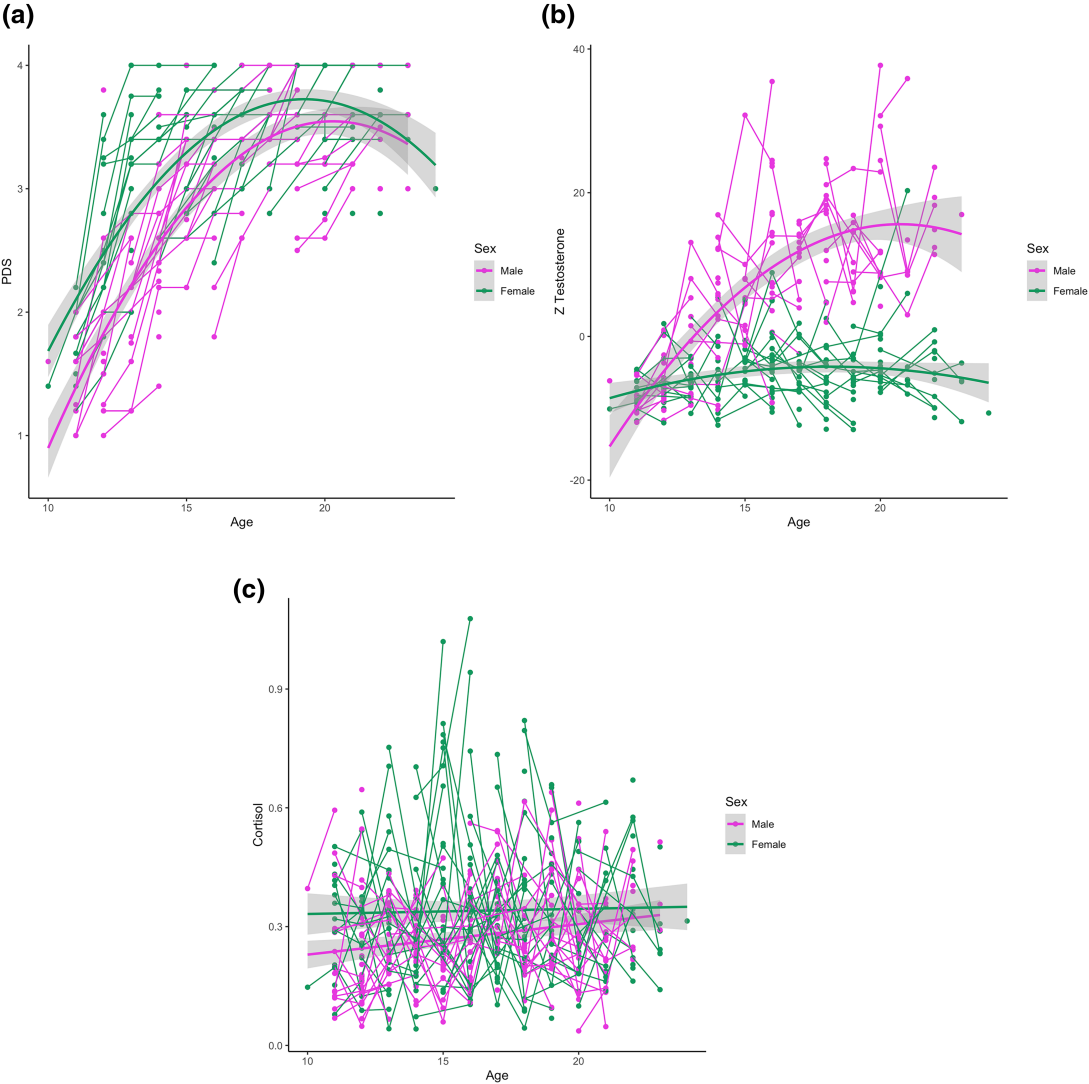
Development of pubertal status (left), testosterone (middle) and cortisol (right) across ages in the SC study. PDS and testosterone showed a quadratic age effect, and no age effect was found for cortisol.

Second, we examined the developmental trajectory of hormone and puberty measures, by testing for associations with age, with sex as an additional factor in the analyses. These findings are reported for the SC study only. An interaction effect was found for sex and quadratic age on PDS (*F*(321.45) = 6.00, *p* = 0.01) and testosterone (*F*(251.74) = 34.32, *p* < 0.001) (Figure 3). PDS showed a steep increase that flattened around age 18 for females and around age 20 for males. Testosterone showed a steep increase for males throughout puberty that flattened around age 20, whereas for females it showed a more gradual increase. No significant age effect for cortisol was found for cortisol (*F*(242.52) = 3.38, *p* = 0.07).

### 3.3 Development of resting‐state connectivity

**FIGURE 4:**
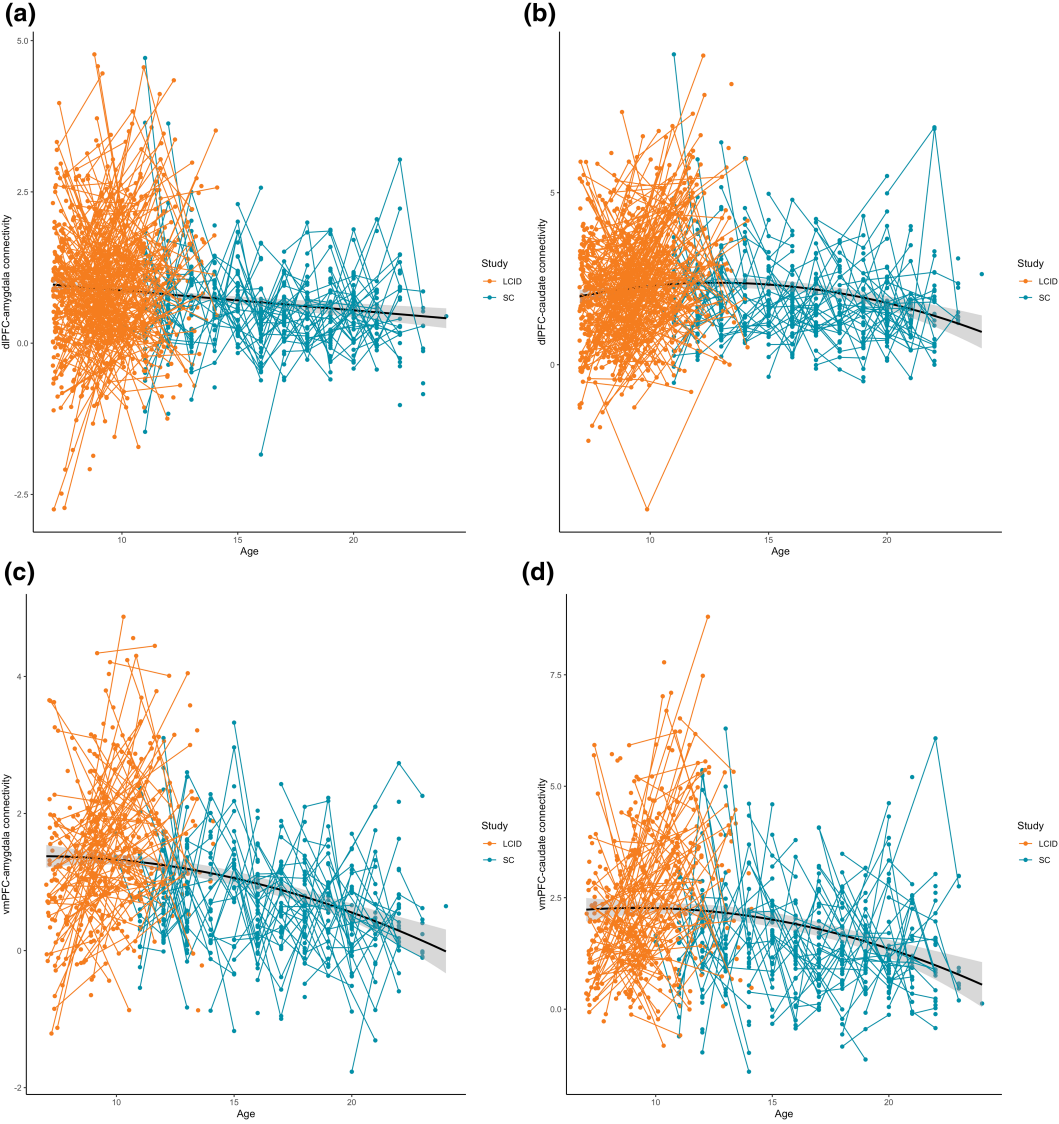
Development of resting state functional connectivity (a: dlPFC–amygdala, b: dlPFC–caudate, c: vmPFC–amygdala, d: vmPFC–caudate) in the combined LCID and SD cohorts. dlPFC–amygdala showed a linear age effect, and the other connections showed a quadratic age effect.

Third, we examined the developmental trajectory of dlPFC–amygdala, dlPFC–caudate, vmPFC–amygdala, and vmPFC–caudate connectivity, by testing for associations with age, with sex as an additional factor in the analyses. These findings are reported for the combined L‐CID and SC cohorts. Connectivity between dlPFC–amygdala showed a linear age effect (*F*(637.52) = 18.80, *p* < 0.001; Figure 4), with a gradual decrease in connectivity (Figure 4a). The dlPFC–caudate, vmPFC–amygdala, vmPFC–caudate connectivity showed quadratic associations with age (*F*(1130.25) = 30.26, *p* < 0.001; *F*(729.98) = 10.71, *p* = 0.001; and *F*(735.49) = 11.95, *p* < 0.001, respectively). DlPFC–caudate connectivity showed a developmental peak with an increase until mid‐adolescence followed by a decrease in late adolescence. In contrast, vmPFC–amygdala and vmPFC–caudate showed a plateau followed by a gradual decrease with age.

### 3.4 Longitudinal associations between pubertal hormones and mood

To investigate the longitudinal within‐person relations between hormone levels (testosterone or cortisol) and mood variability, four RI‐CLPM models were executed. All RI‐CLPM models had an adequate fit and fitted significantly better than the CLPM (Table S2), meaning that the addition of separate modelling of the within‐person effects described the data better. These analyses only included the SC study. No significant autoregressive paths were found in the model with testosterone and mood variability, meaning that within‐person changes in testosterone did not precede future changes in testosterone (*B* = −0.29 to 0.29; Figure S5 and Table S3). In addition, no significant cross‐lagged paths were found in the model with testosterone and mood variability, meaning that within‐person changes in testosterone did not predict within‐person changes in mood variability at a later timepoint, or vice versa.

Next, the same model was tested for cortisol, to examine the longitudinal within‐person relations between cortisol and mood variability. These analyses again only included the SC study. The RI‐CLPM model with cortisol and mood variability resulted in no significant autoregressive or cross‐lagged paths (*B* = −0.19 to 0.14; Figure S6), meaning that within‐person changes in cortisol did not precede within‐person changes in mood variability at a later timepoint or vice versa. The RI‐CLPM fit significantly better than the CLPM (Table S2).

Finally, we tested the same model with pubertal status and mood variability. For the model with pubertal status and mood variability, within‐person PDS at one timepoint significantly predicted within person PDS at the next timepoint (*B* = 0.82 and 0.86, *p* < 0.001; Figure S7), meaning that changes in PDS preceded changes in PDS at the next timepoint. Again, no significant cross‐lagged paths were found, meaning that within‐person changes in PDS did not predict within‐person changes in mood variability at a later timepoint, or vice versa.

**FIGURE 5:**
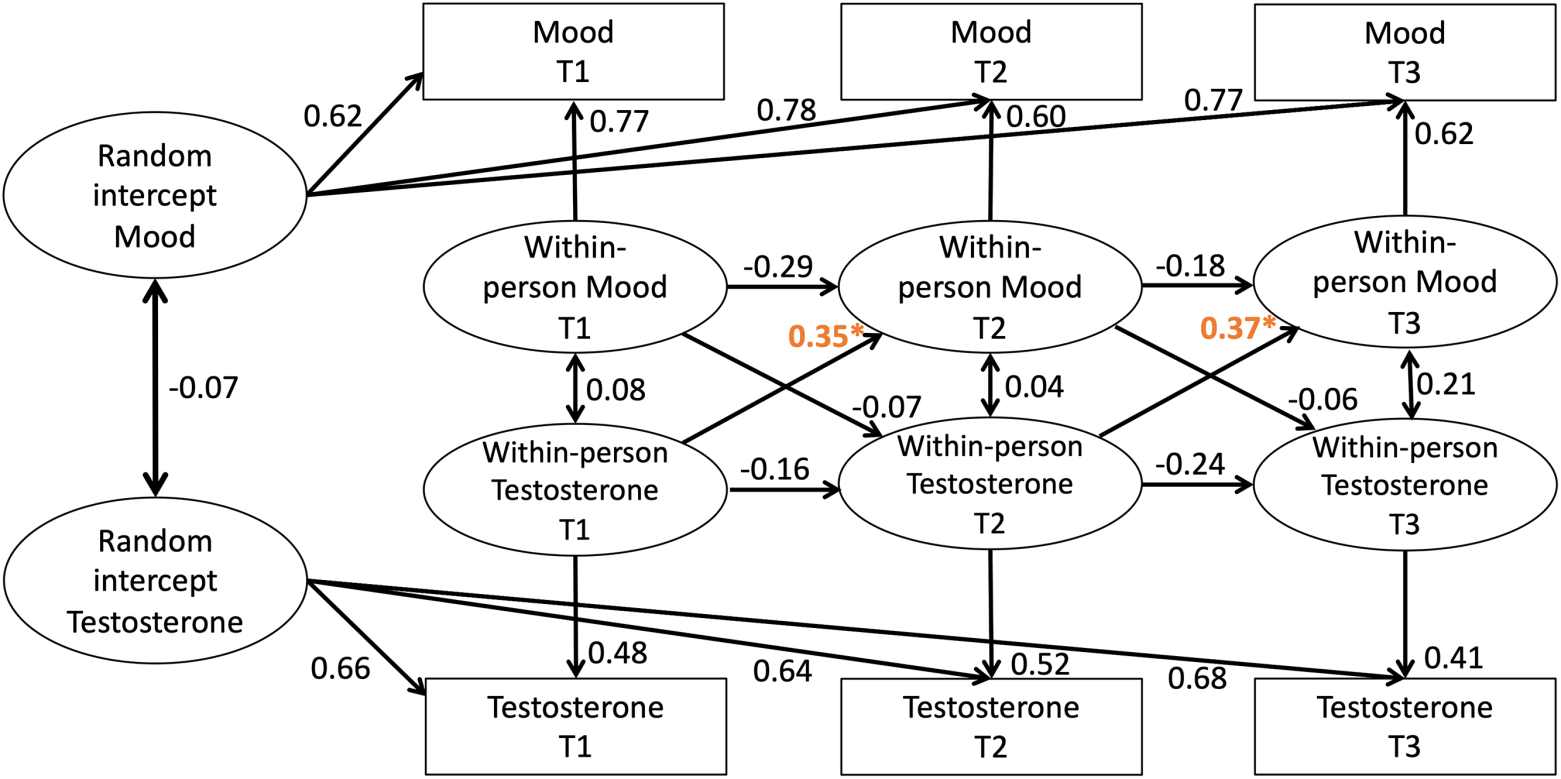
RI‐CLPM with testosterone and mood. Significant cross‐lagged paths were found from testosterone to average mood (indicated by *). Standard estimates are reported.

All these effects were specific for mood variability. Exploratively, the same models were executed with average mood, with similar results with non‐significant cross‐lagged paths, except that significant cross‐lagged paths from testosterone to average mood were found (*B* = 0.35 and 0.37, *p* = 0.05), meaning that within‐person increases in testosterone preceded within‐person increases in mood at a later timepoint (Figure 5).

### 3.5 Resting‐state functional connectivity and mood in the L‐CID and SC cohorts

Next, the within‐person relations between resting state functional connectivity and mood variability were examined in the combined sample of the L‐CID and SC cohorts (Table S2). The RI‐CLPM model with mood variability and dlPFC–amygdala connectivity and with mood variability and dlPFC–caudate showed no significant autoregressive or cross‐lagged paths, meaning that within‐person changes in functional connectivity did not precede within‐person changes in mood variability or functional connectivity at a later timepoint (*B* = −0.18 to 0.25; Figures S8 and S9).

**FIGURE 6:**
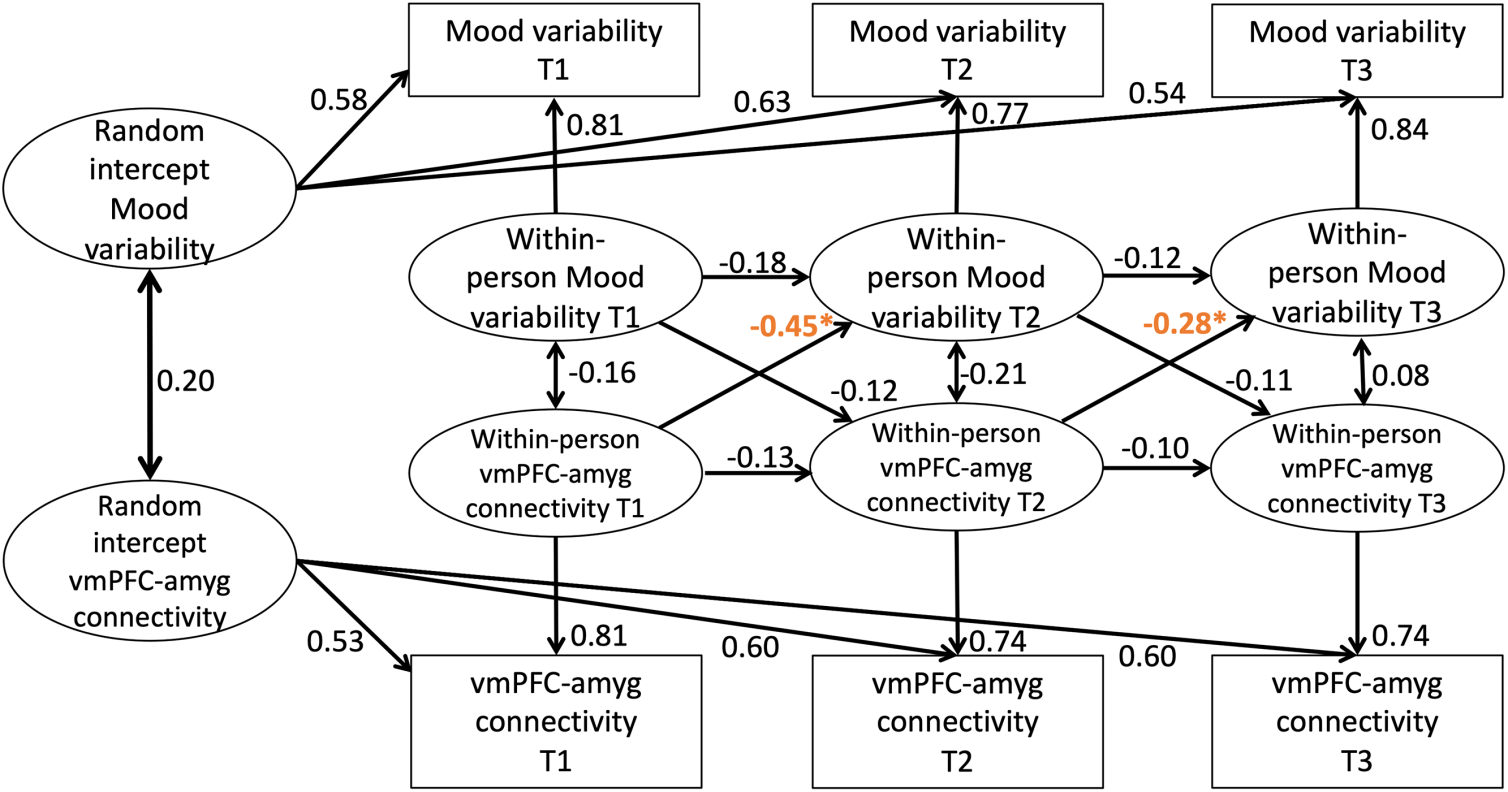
RI‐CLPM with vmPFC–amygdala connectivity and mood variability. Significant cross‐lagged paths from vmPFC–amygdala connectivity to mood variability were found (indicated by *). Standard estimates are reported.

The RI‐CLPM model with vmPFC–amygdala connectivity and mood variability showed significant cross‐lagged paths from vmPFC–amygdala connectivity to mood variability (*B* = −0.45 and −0.28, *p* < 0.001; Figure 6), but no significant autoregressive were found, meaning that within person changes in vmPFC–amygdala connectivity preceded within person changes in mood variability but not vmPFC–amygdala connectivity at a later timepoint. No significant autoregressive or cross‐lagged paths were found for the model with vmPFC–caudate connectivity and mood variability (*B* = −0.28 to 0.05; Figure S10), meaning that within person changes in vmPFC–amygdala connectivity did not precede within person changes in mood variability or vmPFC–amygdala connectivity at a later timepoint.

### 3.6 Hormone levels, resting‐state functional connectivity and mood variability in the SC study

Subsequently, trivariate RI‐CLPMs were used to test whether the relations between hormone levels and mood variability were mediated by brain connectivity (all *p*'s > 0.05). These analyses only included the SC study. No cross‐lagged effects from predictors (hormones) to mediator (brain connectivity) and mediator (brain connectivity) to outcome (mood variability) were found for hormone levels on the relation between resting‐state functional connectivity and mood variability.

## 4 DISCUSSION

The current study aimed to investigate developmental trajectories and within person relations between hormone levels and pubertal status, functional brain connectivity and mood variability during childhood and adolescence using RI‐CLPM. As expected, mood variability peaked in adolescence. Testosterone levels and self‐reported puberty also showed a rapid rise in adolescence (see also Braams et al., 2015). Finally, connections between prefrontal cortex (dlPFC and vmPFC) and subcortical regions (caudate, amygdala) showed patterns of decoupling across development, with both linear and quadratic time courses. When testing for within person relations, we observed two effects: first, testosterone preceded average mood at the next time point, but there was no evidence of puberty development (testosterone, cortisol, or self‐report) preceding mood variability. Second, stronger vmPFC–amygdala resting state functional connectivity preceded more changes in mood variability at the next timepoint. There was no evidence for a longitudinal mediation showing that brain‐connectivity mediated the relation between pubertal measures and mood variability.

### 4.1 Developmental trajectories

This large two‐sample study allowed us to examine developmental time courses of behavioral, biological, and neural measures that have previously been related to mood states in children and adolescents (7–24 years) (Derntl et al., 2009; Uchida et al., 2014; van Honk et al., 1999). Given that participants were included in three waves, this allowed us to fit both the developmental trajectories, as well as the within person stability and variability. We showed the peak of mood variability reported in prior research, but no increase was found in childhood (based on parent‐report), further confirming that entering puberty is associated with a rise in mood variability (Maciejewski et al., 2015; Toenders et al., 2022). In a previous study based on partly the same sample, we also described this adolescent peak in mood variability, which is larger for females than for males (Toenders et al., 2022). The current study further showed that puberty, both self‐report and in terms of testosterone levels, shows a rapid increase in the teenage years (Braams et al., 2015).

### 4.2 Puberty and mood variability

The RI‐CLPM results revealed that within‐person effects were observed for average mood and self‐reported puberty, suggesting person specific developmental trajectories. Contrary to expectations, there were no strong within‐person effects of self‐reported puberty predicting mood variability over time. We did observe a positive within‐person relation between testosterone preceding average mood (not variability) at the next time point. This aligns with previous between‐person and within‐person research linking testosterone with mood (Chronister et al., 2021; Copeland et al., 2019; Culbert et al., 2022; Derntl et al., 2009; Klipker et al., 2017; van Honk et al., 1999). This was found in a combined sample of males and females. It should be noted that prior research reported inconsistencies in the direction of the relation between testosterone and mood in males (Chronister et al., 2021; Copeland et al., 2019; Granger et al., 2003). In the current study we did not examine interactions with sex, however future research should study the relation separately for males and females in larger sample sizes. Testosterone did not show within‐person stability, showing that testosterone within the individual levels are variable over time, so possibly the effects of testosterone on mood found in prior research are through stimulating other systems in the brain. The mechanism of action of testosterone on mood might be through androgen receptors in the brain. In rats it has been shown that through these androgen receptors, testosterone can affect dendritic growth, myelination, and density of dendritic synapses, all factors that contribute to the ongoing maturation of the brain during adolescence (Abi Ghanem et al., 2017; Goldstein et al., 1990; Leranth et al., 2003). Androgen receptors are found in the hippocampus and the cortex, including the dlPFC, OFC, hypothalamus, amygdala, and visual cortex (Clark et al., 1988), and their expression in rats increased with age (Kerr et al., 1995). These effects should be investigated in more detail in future research.

An unexpected finding was the absence of a predictive within‐person relations between testosterone, cortisol, and mood. Previous studies showed a relation between cortisol and mood variability in adults in a between‐person design (Human et al., 2015; Simpson et al., 2008), but we were unable to find such results or within‐person relations in children and adolescents. Hormone levels are known to fluctuate (Ross et al., 2014), and previous studies have studied the relation with mood measured at the same time. The efficacy of hormone levels is short‐lived and does not sustain over the course of a year, especially in developing adolescents. Future studies should investigate the within‐person associations in a more fine‐grained timeframe and include other pubertal hormones such as estradiol to be able to measure larger variability in pubertal hormones in females (Balzer et al., 2015). Additionally, cortisol might show more of an association with trait levels of mental health, instead of mood state and mood variability (Van den Bergh et al., 2008).

### 4.3 Cortical–subcortical resting state and mood variability

The next question that was addressed in this study was the relation between cortical–subcortical resting state connectivity and mood variability. First, we found decoupling between dlFPC and amydala over time, in contrast to what we expected based on prior research that showed an age‐related increase in connectivity between the dlPFC and subcortical regions (van Duijvenvoorde et al., 2016). However, the amygdala specifically, has also been shown to have a age‐related decrease in connectivity to the PFC (Xiao et al., 2018). Second, we observed a quadratic pattern for dlPFC–caudate connectivity, with an increase in connectivity followed by a decrease in connectivity. The caudate is part of the striatum and has been of much interested in adolescent reward models, which reported adolescent‐specific peaks in neural activity in the striatum in the context of reward processing (Silverman et al., 2015). This study shows that also connectivity between the dlPFC and striatum may peak in adolescence, possibly indicating a sensitive window for goal motivation (Luna et al., 2013). Finally, connectivity between vmPFC–amydala and vmPFC–caudate also showed age‐related decoupling with a plateau in childhood and a rapid decrease in connectivity in adolescence (van Duijvenvoorde et al., 2016).

When testing for time dependent relations, we observed negative within‐person relation between vmPFC–amygdala resting state connectivity and mood variability. The vmPFC is involved in implicit emotion regulation (Etkin et al., 2015), and connectivity between the vmPFC and amygdala has been previously associated with emotion regulation (Perlman & Pelphrey, 2011). It could be speculated that within person change (more development) in the connectivity between these regions, might lead to less within‐person change mood through emotion regulation. Surprisingly, no within‐person association between dlPFC to subcortical connectivity and mood was found. Even though the dlPFC–amygdala connection has also been associated with the regulation of emotions (Berboth & Morawetz, 2021; Kroes et al., 2019).

It should be noted that prior research is not conclusive on the direction of the relation between brain connectivity and emotion regulation. Other research showed, using an emotion regulation task, that higher amygdala to ventral lateral PFC (vlPFC) connectivity was associated with successful emotion regulation, and less connectivity was associated with higher levels of anxiety and depression in a transdiagnostic sample (Morawetz et al., 2016). In line, a rs‐fMRI study that used a self‐report questionnaire to report emotion regulation difficulties reported that impaired emotion regulation was associated with stronger negative connectivity between the amygdala and prefrontal regions (Rabany et al., 2017). Hence, task‐based functional MRI studies suggest that greater coupling between the amygdala and prefrontal regions is associated with emotion regulation but resting state studies show inconclusive results (Berboth & Morawetz, 2021). The current study expanded on the previous research by showing a longitudinal within‐person association between cortical and subcortical areas (vmPFC–amygdala) and mood states over a period of a year, suggesting that the development of brain connectivity might predict future mood.

### 4.4 Puberty, brain connectivity, and mood

The final goal of this study was to test a mediation model to examine whether brain connectivity affected the relation between puberty and mood variability. However, no evidence for these relations was found. Possibly, our measures were limited to observe these relations over time, also given that our measures for mood variability were relatively limited in scope and we did not test for mental health outcomes. In prior research, mood variability has previously been shown to precede future symptoms of anxiety and depression (Maciejewski et al., 2015; Toenders et al., 2022), which implies that mood variability can have long‐term implications. Since these studies focused on between‐person effects, it could be that on a group level mood variability precedes mental ill‐health, but on an individual level mood variability is highly variable.

### 4.5 Strengths, limitations, and future directions

Several strengths and limitations of the study should be noted. By using models with random intercepts, we decomposed the differences between individuals from differences within individuals. Given that the two included cohorts were scanned using the same facilities and the same protocols, this provided us with the unique possibility to map the developmental time course from childhood (7–14 years) to adulthood (11–24 years) with a significant sample size. The inclusion of young children allowed us to observe trends that were not yet observed in prior research. Nonetheless, some limitations should be acknowledged. Although a developmental sample was studied, the model in combination with the study design did now allow us to examine within‐person relations in specific developmental timeframes (e.g., the within‐person between testosterone and mood variability in early adolescence only). Thus, no conclusions can be drawn about a specific developmental period where one might be more susceptible for deviations in brain connectivity. Therefore, future studies should study the within‐person associations in large cohort studies, that allow for the testing of within‐person effects in specific developmental timeframes. In addition, two different approaches were used to assess mood variability in the separate studies, which might have affected the results. SC used a self‐report, while L‐CID used a parent‐reported measure of mood, because of the age of the participants. However, this could also be seen as a strength as it might allow us to rule out the role of emotional awareness (Subic‐Wrana et al., 2014). The within‐person relation between vmPFC–amygdala connectivity and mood variability was found across the two cohorts using different measures of mood, which suggests that the association is not merely explained by the participants being better at emotion recognition but this connection being involved in mood variability (Stranger & Lewis, 1993; Stringaris & Goodman, 2009). To be able to combine datasets, which is especially important given the large amount of data needed to study replicable brain‐behavior associations (Marek et al., 2022), it is important to create one gold standard to measure mood variability. This measure should consider the burden for participants to fill out questionnaires and the reliability of a self vs parent‐report. Third, we conducted multiple RI‐CLPMs, which increases the risk for false positive findings. As this is an exploratory study for within‐person effects between mood variability and biological factors, future studies are needed to replicate these results. Lastly, due to the sample size this study did not examine the within‐person relations separately per sex, but solely controlled for sex in the analyses. However, mood and mood variability develop differently in females and males (Toenders et al., 2022), which is underscored by the differences in prevalence in mental ill‐health between females and males (Kieling et al., 2024). Pubertal hormone development also shows strong sex effects (Handelsman et al., 2016), whereas only specific resting state connections show these sex effects (van Duijvenvoorde et al., 2019). Thus, these biological measures might explain the sex‐specific development of mood swings. The current study did not have the power to test for relations in males and females separately but controlled for sex instead (Mulder, 2022). Future, larger, studies should further examine possible sex differences in mood variability and its biological mechanisms to disentangle these effects and study the effects of other pubertal hormones more relevant to females such as estradiol. Finally, it is important to consider both sex and gender effects as they might affect experiences of mood differently.

### 4.6 Conclusions

To conclude, the current study confirmed the hypothesis that within‐person vmPFC–amygdala connectivity, important for emotion regulation, predicted future mood variability during childhood and adolescence. In addition, within‐person testosterone predicted average mood at the next time point. These within‐person effects show that puberty and brain connectivity are involved in the development of mood (variability) in adolescence. Future studies are needed to further explore the extent of this relation and how this plays a role in the development of emotion regulation. With this study we show that brain connectivity during development is an important biological mechanism of the development of mood in young people.

## CONFLICT OF INTEREST STATEMENT

The authors declare no conflicts of interest.

## Supporting information


**Data S1.** Supporting Information.

## Data Availability

The data used is the study is available from the corresponding author upon request with a formal data sharing agreement. The code will be made available in the EUR data repository upon publication.
