# Supplementary material for: Within‐person biological mechanisms of mood variability in childhood and adolescence
Source: Hum Brain Mapp. 2024 Jul 24;45(11):e26766. doi: 10.1002/hbm.26766 (PMC11267453; doi:10.1002/hbm.26766)
Supplement: Supplementary file 1 — Data S1. Supporting Information. [file HBM-45-e26766-s001.docx]

**Supplementary Information**

**
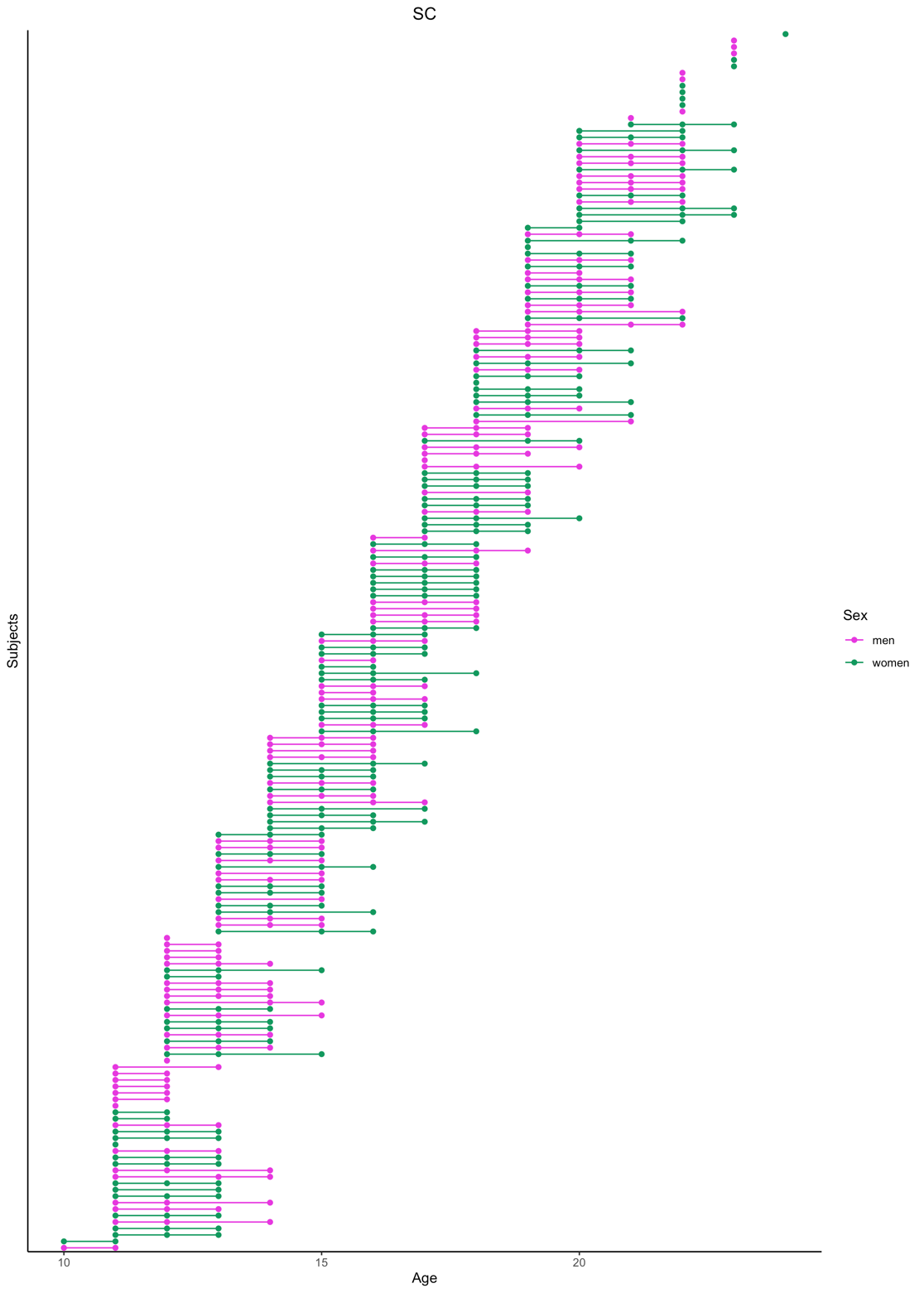
**

**Supplementary Figure S1. Age per subject in SC**

**
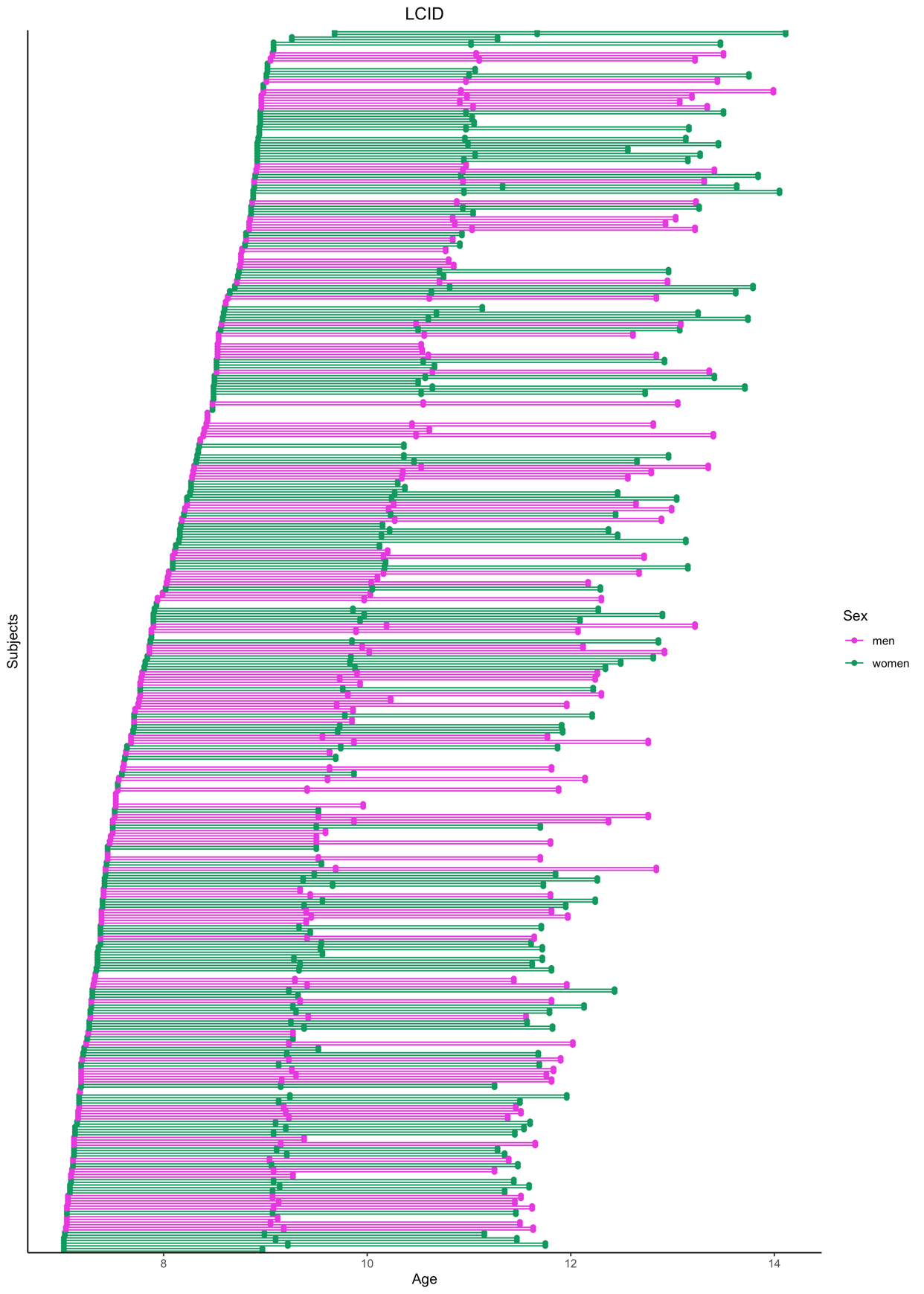
**

**Supplementary Figure S2. Age per subject in LCID**

**
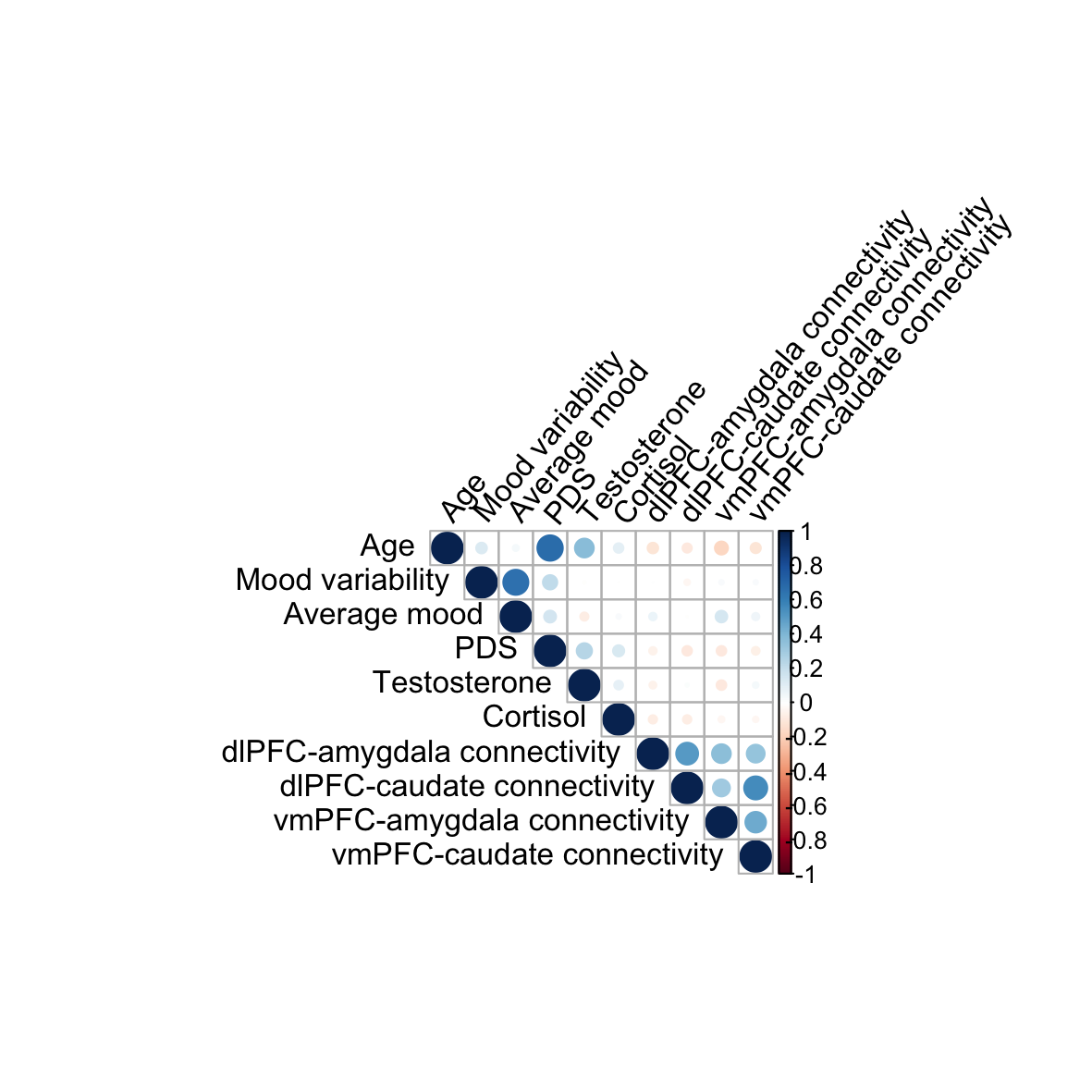
**

**Supplementary Figure S3. Correlation matrix SC study**

**
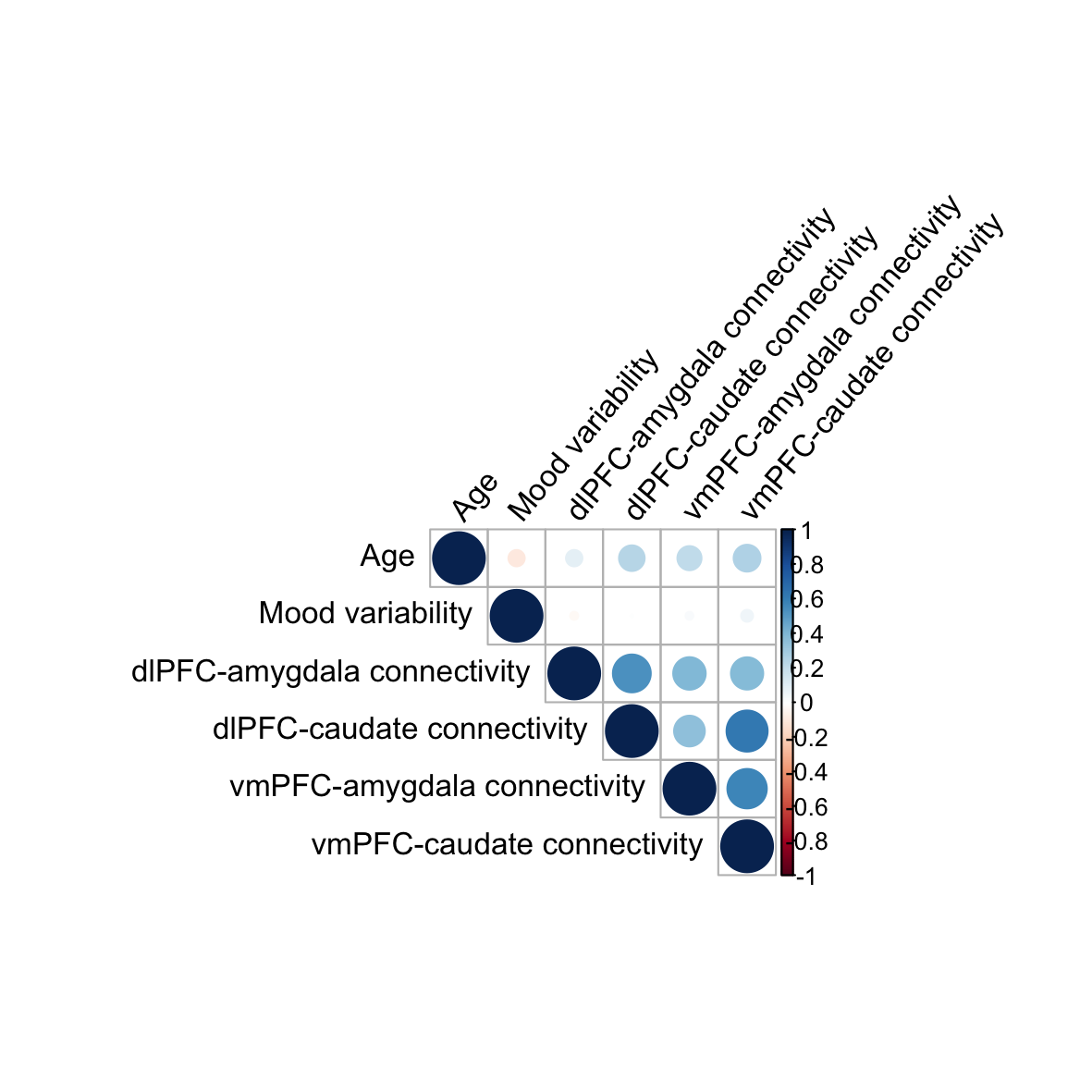
**

**Supplementary Figure S4. Correlation matrix LCID study**

**Supplementary Table S1. Correlation within outcomes between timepoints**

|  | **SC** | | |
| --- | --- | --- | --- |
|  | Timepoint 1 – Timepoint 2 | Timepoint 2 – Timepoint 3 | Timepoint 1 – Timepoint 3 |
| Average Mood | 0.412* | 0.677* | 0.282* |
| Mood variability | 0.261* | 0.437* | 0.413* |
| Testosterone | 0.694* | 0.751* | 0.796* |
| Cortisol | 0.184 | 0.219 | 0.073 |
| PDS | 0.878* | 0.951* | 0.790* |
| Connectivity dlPFC – amygdala | 0.308* | 0.158 | 0.220 |
| Connectivity dlPFC – caudate | 0.501* | 0.085 | 0.355* |
| Connectivity vmPFC – amygdala | 0.249* | 0.253* | 0.254 |
| Connectivity vmPFC – caudate | 0.306* | 0.319* | 0.422* |
|  | **L-CID** | | |
|  | Timepoint 1 – Timepoint 3 | Timepoint 3 – Timepoint 5 | Timepoint 1 – Timepoint 5 |
| Mood variability | 0.221* | 0.210* | 0.220* |
| Connectivity dlPFC – amygdala | 0.422* | 0.392* | 0.311* |
| Connectivity dlPFC – caudate | 0.305* | 0.161* | 0.320* |
| Connectivity vmPFC – amygdala | 0.276 | 0.579* | -0.383* |
| Connectivity vmPFC – caudate | 0.314 | 0.687 | 0.646 |

**Supplemental Table S2. Fit measures of RI-CLPM and comparison to CLPM**

| Variable of interest | Mood variable | χ2 (df) | *p* | CFI | RMSEA | ΔS-B χ2 (df) | *p* |
| --- | --- | --- | --- | --- | --- | --- | --- |
| Testosterone | Mood variability | 6.79 (9) | .66 | 1.00 | .00 | 31.76 (3) | <.001 |
| Testosterone | Average mood | 6.17 (9) | .72 | 1.00 | .00 | 195.32 (3) | <.001 |
| Cortisol | Mood variability | 3.81 (9) | .92 | 1.00 | .00 | 8.26 (3) | .030 |
| Cortisol | Average mood | 7.52 (9) | .58 | 1.00 | .00 | 40.03 (3) | <.001 |
| PDS | Mood variability | 9.05 (9) | .43 | 1.00 | .01 | 14.04 (3) | .003 |
| PDS | Average mood | 16.03 (9) | .07 | .98 | .07 | 11.19 (3) | .011 |
| dlPFC- amygdala connectivity | Mood variability | 24.60 (15) | .06 | .83 | .04 | 8.24 (3) | .041 |
| dlPFC- caudate connectivity | Mood variability | 46.74 (15) | <.001 | .64 | .07 | 11.89 (3) | .008 |
| vmPFC- amygdala connectivity | Mood variability | 28.79 (15) | .02 | .84 | .06 | 16.92 (3) | <.001 |
| vmPFC- caudate connectivity | Mood variability | 32.22 (15) | .006 | .83 | .06 | 12.60 (3) | .006 |

**Supplemental Table S3.** **Model characteristics RI-CLPM mood variability**

|  | RIx-RIy | wx1-wy1 | .wx2-.wy2 | .wx3-.wy3 |
| --- | --- | --- | --- | --- |
| Testosterone and mood variability | .15 | .17 | .09 | -0.05 |
| Cortisol and mood variability | -.08 | -.08 | -.01 | -.02 |
| PDS and mood variability | -.08 | .30 | .28 | .07 |
| dlPFC- amygdala connectivity and mood variability | -.06 | -.04 | .03 | .14 |
| dlPFC- caudate connectivity and mood variability | -.14 | .05 | .04 | -.01 |
| vmPFC- amygdala connectivity and mood variability | .20 | -.16 | -.21 | .08 |
| vmPFC- caudate connectivity and mood variability | .16 | -.08 | -.13 | .13 |

RIx-RIy: correlation between random intercepts (between-person); wx1-wy1: correlation between within-person changes at T1; wx2-wy2: correlation between within-person changes at T2; wx3-wy3: correlation between within-person changes at T3

**
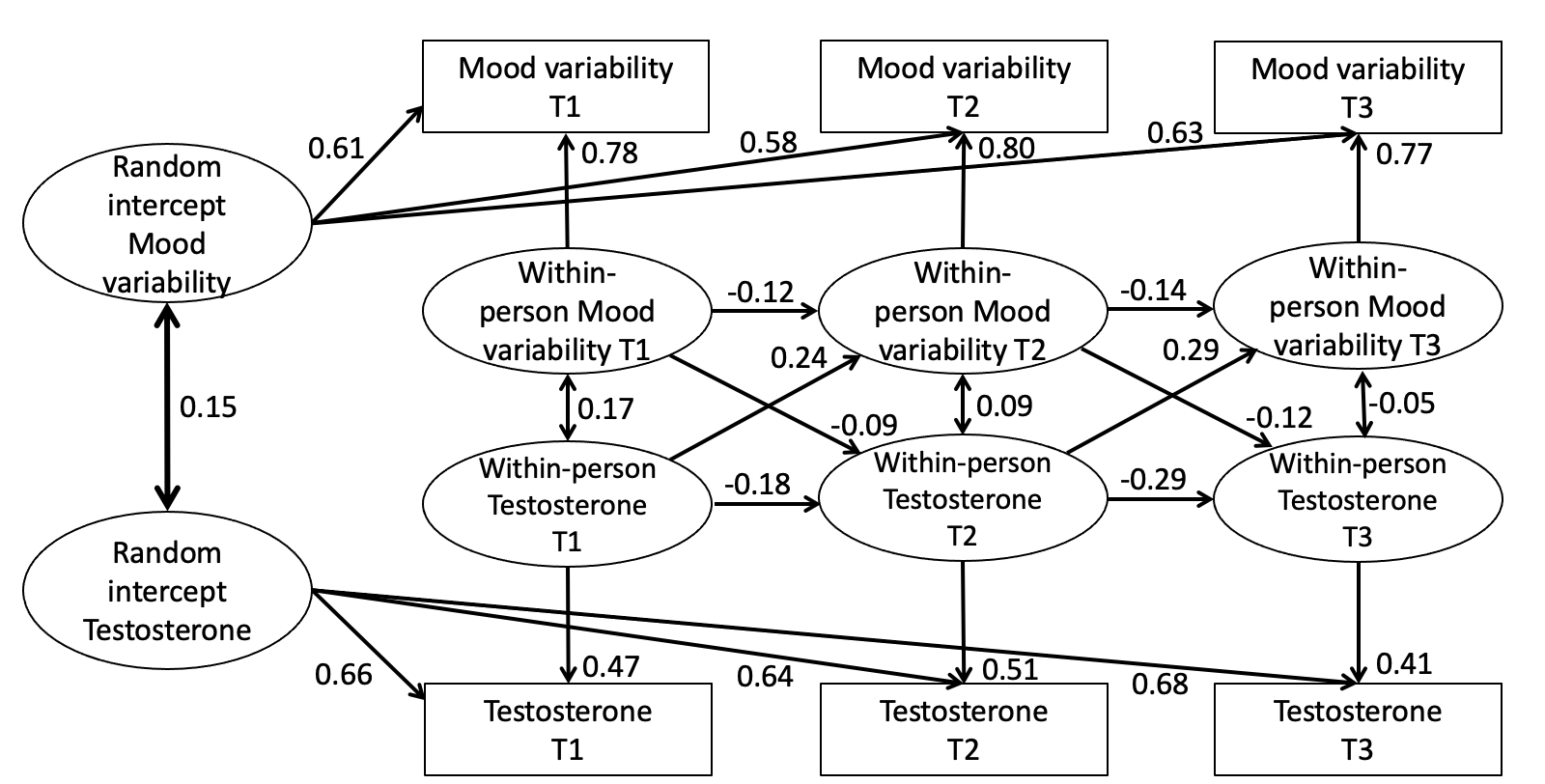
**

**Supplementary Figure S5. RI-CLPM with testosterone and mood variability.** No significant auto-regressive or cross-lagged paths were found.

**
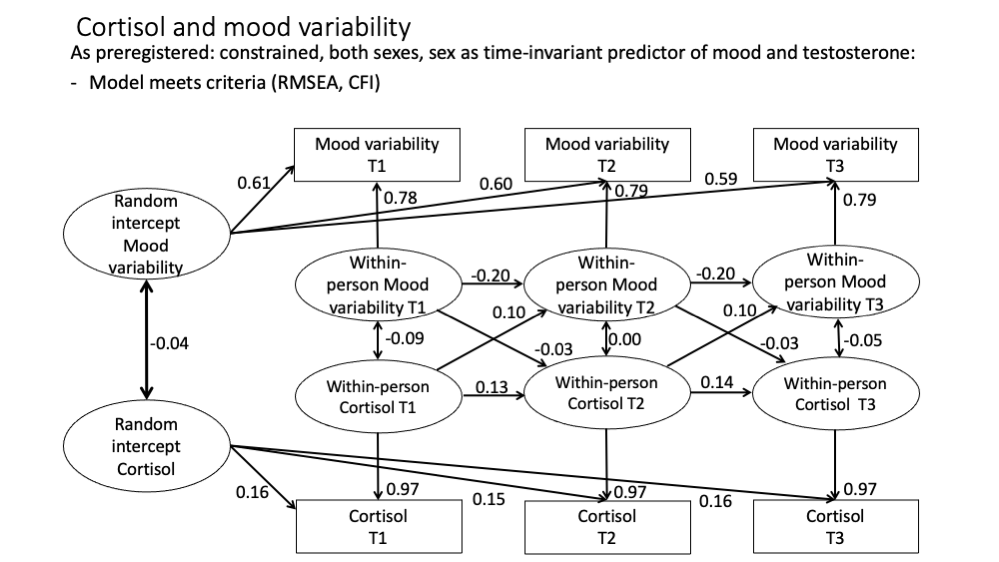
**

**Supplementary Figure S6. RI-CLPM with cortisol and mood variability.** No significant auto-regressive or cross-lagged paths were found.

**
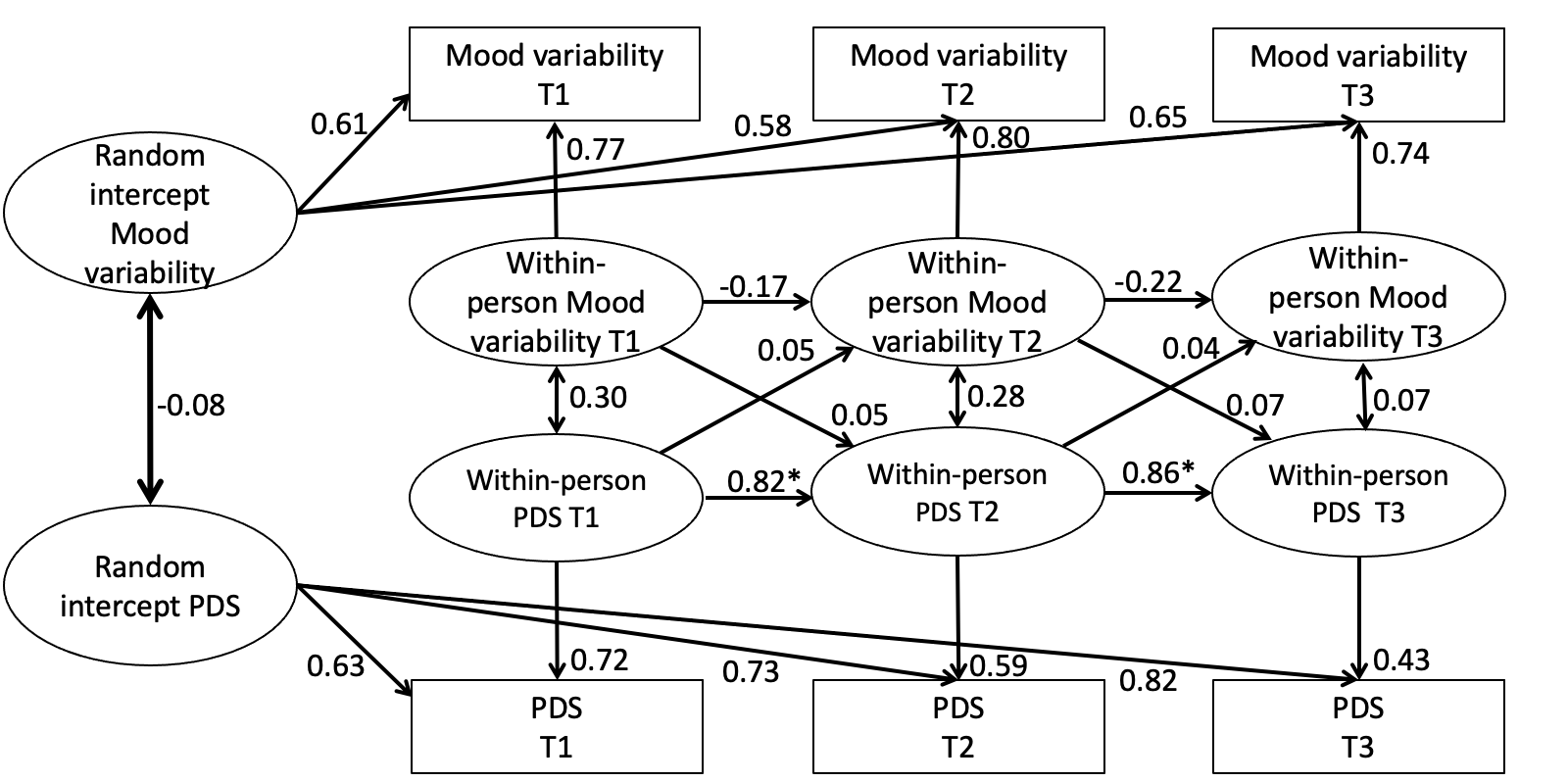
**

**Supplementary Figure S7. RI-CLPM with PDS and mood variability.** Significant auto-regressive paths for PDS were found, but not for mood variability, and no significant cross-lagged paths were found.

**
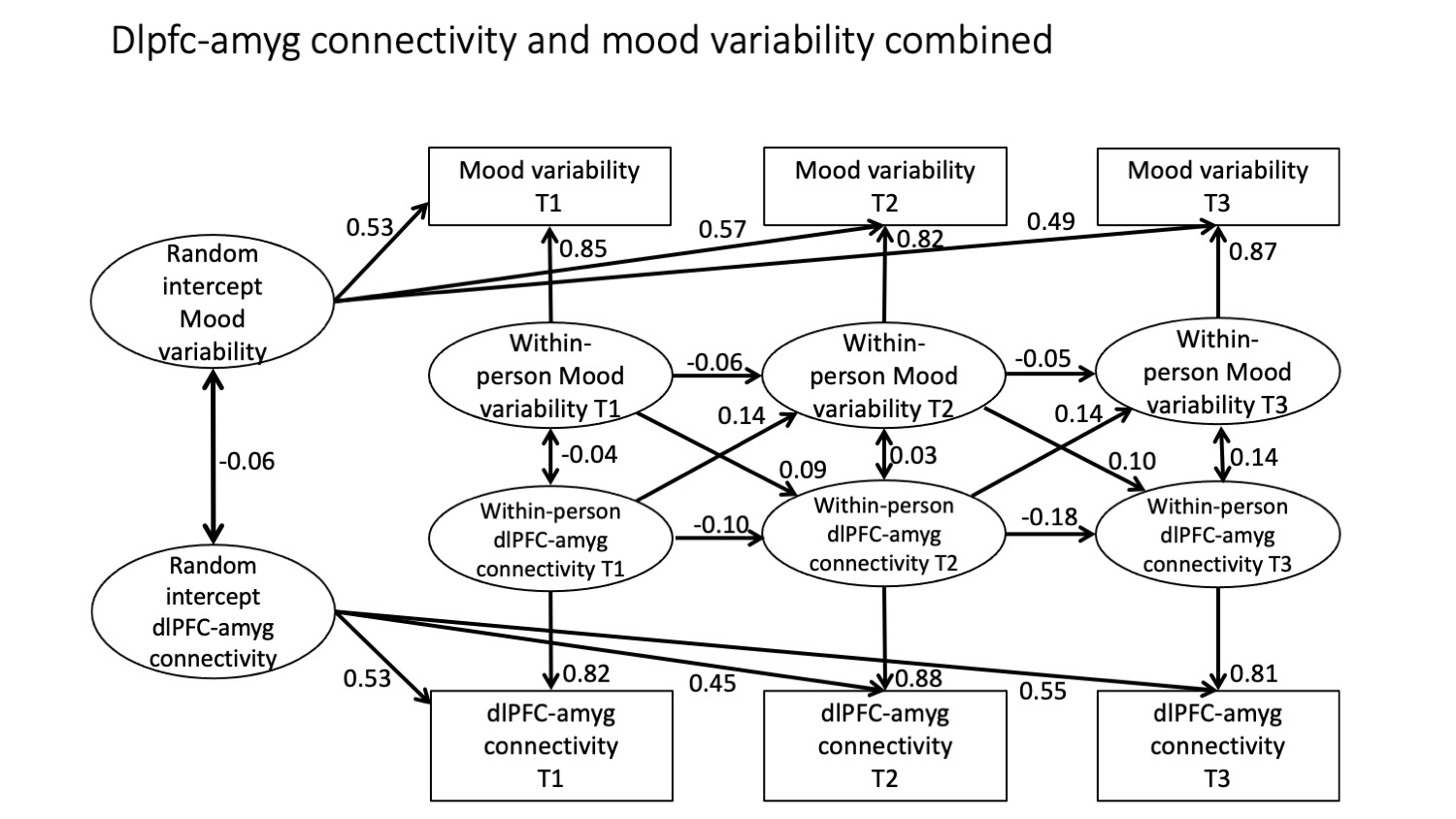
**

**Supplementary Figure S8. RI-CLPM with dlPFC-amygdala connectivity and mood variability.** No significant auto-regressive or cross-lagged paths were found.

**
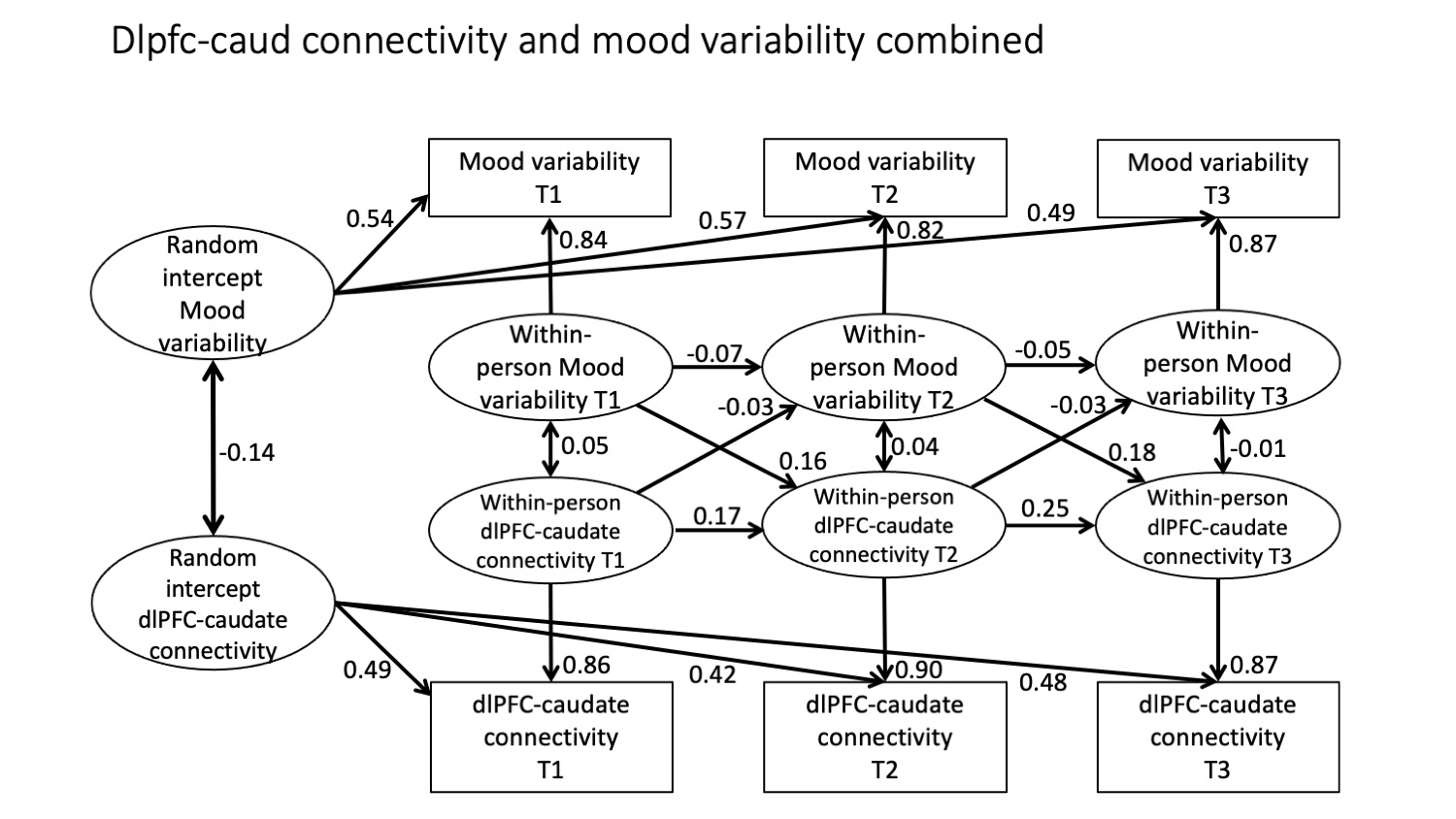
**

**Supplementary Figure S9. RI-CLPM with dlPFC-caudate connectivity and mood variability.** No significant auto-regressive or cross-lagged paths were found.

**
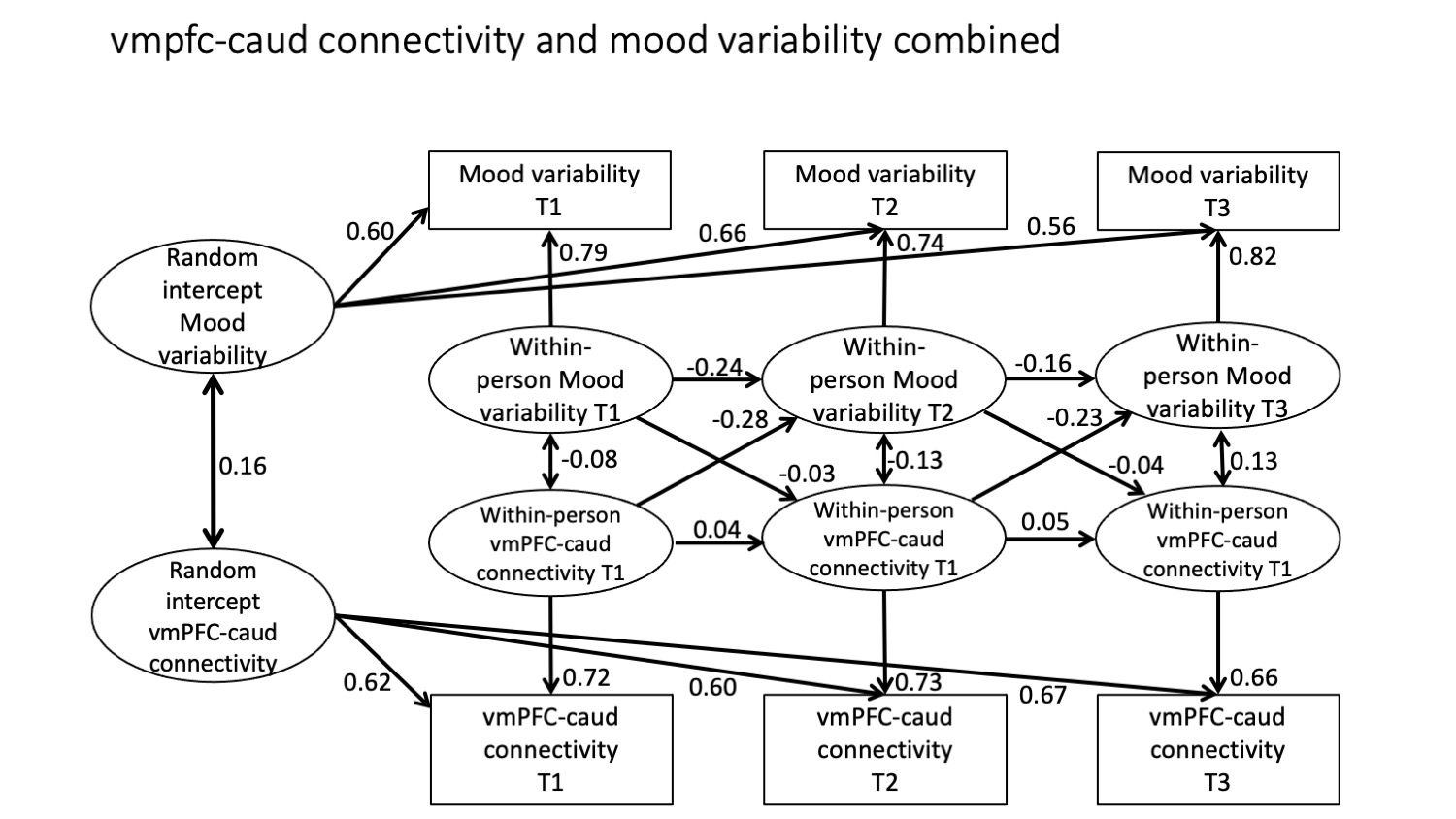
**

**Supplementary Figure S10. RI-CLPM with vmPFC-caudate connectivity and mood variability.** No significant auto-regressive or cross-lagged paths were found.
